# RFE-YOLO: A Study on Photovoltaic Module Fault Detection Algorithm Based on Multimodal Feature Fusion

**DOI:** 10.3390/s25216774

**Published:** 2025-11-05

**Authors:** Yuyang Guo, Xiuling Wang, Zhichao Lin

**Affiliations:** 1 College of Information Engineering, Inner Mongolia University of Technology, Hohhot 010080, China; 20241800088@imut.edu.cn (Y.G.);; 2 Inner Mongolia Key Laboratory of Perceptive Technology and Intelligent Systems, Hohhot 010080, China

**Keywords:** photovoltaic power plant, fault detection, YOLOv11, multimodal images, feature fusion

## Abstract

The operational status of photovoltaic modules directly impacts power generation efficiency, making rapid and precise fault detection crucial for intelligent operation and maintenance of Photovoltaic (PV) power plants. Addressing the perceptual limitations of single-modal images in complex environments, this study constructs an RGBIRPV multimodal dataset tailored for centralized PV power plants and proposes an RFE-YOLO model. This model enhances detection performance through three core mechanisms: The RC module employs a CBAM-based attention mechanism for multi-parameter feature extraction, utilizing heterogeneous RC_V and RC_I architectures to achieve differentiated feature enhancement for visible and infrared modalities. The lightweight adaptive fusion FA module introduces learnable modality balance and attention cascading mechanisms to optimize multimodal information fusion. Concurrently, the multi-scale enhanced EVG module based on GSConv achieves synergistic representation of shallow details and deep semantics with low computational overhead. The experiment employed an 8:1:1 data partitioning scheme. Compared to the YOLOv11n model employing feature-level mid-fusion, the model proposed in this study achieves improvements of 2.9%, 1.8%, and 1.5% in precision, mAP@50, and F1 score, respectively. It effectively meets the demand for rapid and accurate detection of PV module failures in real power plant environments, providing an effective technical solution for intelligent operation and maintenance of photovoltaic power plants.

## 1. Introduction

With the increasingly severe global climate change challenges, the demand for energy transition has become increasingly urgent. Solar photovoltaic power generation, as one of the most promising renewable energy technologies, is experiencing rapid development worldwide [1,2]. According to the latest data from the International Renewable Energy Agency, global cumulative PV installed capacity exceeded 2.2 TW by the end of 2024, representing significant growth compared to 1.6 TW in 2023, with annual new installations exceeding 600 GW. It is projected that between 2024 and 2030, the world will add over 5500 GW of renewable energy capacity, with its share in global power generation capacity exceeding 20%. Centralized PV power plants have become the primary driving force in the PV industry development due to their economies of scale and cost advantages, with individual installation capacities evolving from tens of megawatts in the early stages to hundreds of megawatts and even gigawatt-scale at present. However, as PV power plant scales continue to expand and operational lifespans increase, PV module failure issues have become increasingly prominent, constituting a critical bottleneck constraining the sustainable development of the PV industry. Real-time fault detection and classification in photovoltaic systems represents a fundamental task for ensuring power plant operational quality [3]. During long-term outdoor operation, PV modules are subjected to the combined effects of multiple environmental factors, including ultraviolet radiation, temperature cycling, humid-heat environments, wind-sand erosion, and mechanical stress, making them susceptible to various failures and performance degradation [4,5]. The primary failure modes include hot-spot effects, cell cracks, backsheet degradation, junction box failures, and soiling, with their occurrence mechanisms closely related to installation types, geographical locations, and environmental conditions [6]. These failures not only result in a 5–25% reduction in module power output but may also trigger serious safety incidents such as arcing and fires in severe cases, causing substantial economic losses and safety hazards [7].

Traditional photovoltaic power plant operation and maintenance primarily rely on three approaches: manual inspection, electrical parameter monitoring through supervisory systems, and ground-based robotic inspection. Manual inspection requires O&M personnel to carry handheld infrared thermal cameras or multimeters to examine PV modules individually. While this method can identify certain obvious appearance defects and electrical faults, it exhibits significant limitations. First, the detection efficiency is extremely low. For a 300 MW photovoltaic power plant containing approximately one million modules, comprehensive manual inspection often requires several months to complete [8]. Second, the detection costs are prohibitively high, necessitating substantial human resource investment, with annual O&M costs reaching 2–3% of the total plant investment [9]. Additionally, maintenance personnel must operate in harsh environments characterized by high temperatures, high voltages, and complex terrain, facing significant safety risks including electrical shock and falls [10]. More critically, manual inspection is susceptible to subjective factors such as personnel skill levels and working conditions, leading to quality issues including missed detections and false positives, which severely compromise the reliability and practicality of manual inspection approaches [11].

Although electrical parameter monitoring based on supervisory systems enables remote automated monitoring, it can only monitor electrical parameter variations at the string or inverter level, failing to precisely locate specific faulty components and exhibiting insufficient sensitivity to early-stage faults. Al-Subhi et al. proposed a universal photovoltaic cell parameter estimation method based on optimized deep neural networks, achieving adaptive parameter identification across temperature, irradiance, and power rating variations, thereby providing a new paradigm for precise modeling of intelligent PV systems [12]. Satpathy et al. developed a low-cost monitoring system based on power line communication, integrating optimized sensor networks with web applications to achieve real-time performance monitoring, multi-type fault diagnosis, and alarm functions [13]. When electrical parameters exhibit anomalies, faults have typically progressed to severe stages, missing the optimal maintenance window and potentially triggering safety incidents such as fires [14]. Furthermore, electrical monitoring cannot identify faults that do not affect short-term electrical performance but may lead to long-term reliability issues.

Ground-based robotic inspection, as an emerging automated inspection method in recent years, has partially addressed the issues of low efficiency in manual inspection and insufficient precision in electrical monitoring. Ground inspection robots typically employ wheeled or tracked mobile platforms equipped with visible light cameras, thermal infrared cameras, LiDAR, and other sensor equipment, enabling autonomous navigation along predetermined paths while collecting photovoltaic module images. Compared to manual inspection, ground-based robots offer advantages including 24 h continuous operation capability, standardized detection protocols, and immunity to subjective human factors. Although ground-based robotic inspection has improved detection efficiency to some extent, it still exhibits numerous limitations. The mobility of robots is severely constrained, as they can only traverse ground roads or pathways between modules. When confronting complex terrain conditions and dense module layouts in large-scale photovoltaic power plants, robots often fail to reach all inspection positions. There remains considerable room for technological advancement in autonomous route planning and navigation, as well as photovoltaic fault detection and feature analysis [15]. Furthermore, ground-based robots suffer from limited detection perspectives, capable of capturing module images only from lateral or oblique downward angles, failing to obtain frontal views of module surfaces, which directly compromises fault detection accuracy and reliability. The environmental adaptability of robots is also suboptimal, as they are prone to mobility difficulties or equipment failures under adverse conditions such as rain, snow, waterlogged surfaces, or loose sandy terrain, severely limiting their application effectiveness in complex environments. Additionally, ground-based robots require regular maintenance of critical components, including tires, batteries, and sensors, resulting in increased maintenance frequency and persistently high operational costs [16]. While ground-based robotic detection efficiency shows improvement compared to manual inspection, it remains significantly inferior to the large-scale and terrain-independent coverage capabilities of unmanned aerial vehicles, which, despite also relying on batteries and optical sensors, offer higher flexibility and operational efficiency.

With the rapid development of unmanned aerial vehicle (UAV) remote sensing technology, its applications in the renewable energy sector have become increasingly widespread, particularly demonstrating significant advantages in photovoltaic power plant inspection and fault diagnosis. UAVs possess characteristics of high efficiency, flexibility, and low cost, enabling them to carry various sensors and complete image acquisition of large-area photovoltaic modules within short timeframes. This study constructs a dual-modal image dataset for photovoltaic module fault detection, with data sourced from fixed-altitude aerial photography conducted by UAVs over photovoltaic power plants, encompassing both visible light and thermal infrared modalities. The dataset focuses on three common and representative fault types: hot spots, diode short circuits, and physical obstructions. Unlike traditional single-modal images, dual-modal data can more comprehensively reflect the optical and thermal characteristics of module surfaces. Hot spot regions typically manifest as distinct high-temperature concentration points in infrared images while potentially lacking obvious features in visible light images. Diode short circuits form regularly distributed abnormal thermal zones in infrared images, often accompanied by localized temperature increases. Physical obstructions are primarily evident in visible light images, such as birds, fallen leaves, or foreign objects covering panel surfaces, affecting light absorption. These faults exhibit characteristics including significant inter-modal presentation differences and large variations in regional dimensions. Particularly, some hot spot or obstruction areas occupy only minimal proportions of the module surface, further increasing detection difficulty.

**Motivation and Research Contributions**: Photovoltaic modules in actual operation are susceptible to faults, including hot spots, diode short circuits, and physical obstructions. These fault types exhibit high diversity and complexity, with significantly different manifestations in images, presenting enormous challenges for automated detection. Hot spots are typically small in volume and irregularly distributed, often difficult to identify in visible light images, while presenting only weak temperature anomalies in infrared images. Diode short circuits, although possessing certain thermal characteristics, exhibit variable patterns and are easily confused with other thermal anomalies. Physical obstructions often have irregular shapes and uncertain locations, appearing only in visible light images. To address these challenges, this study proposes a multimodal data-based fault detection method for photovoltaic modules. The method employs a specially designed YOLOv11 model architecture capable of accurately identifying typical fault patterns in photovoltaic modules, including hot spots, diode short circuits, and physical obstructions. Through preprocessing and size standardization of dual-modal images (visible light and thermal infrared) collected by UAVs, the RGBIRPV dataset was constructed. Based on a dual-branch feature extraction structure and cross-modal fusion module, the proposed YOLOv11 model achieves high-precision detection of dual-modal images, demonstrating excellent performance in fault recognition of hot spots, diode short circuits, and obstructions. The proposed end-to-end detection pipeline not only significantly improves the accuracy and recall rate of fault identification but also provides robust technical support for the refined and high-frequency deployment of intelligent operation and maintenance systems in photovoltaic power plants. The effectiveness of this method establishes a solid foundation for the practical and large-scale application of UAV-based automatic inspection technology for photovoltaic modules.

## 2. Related Work

With the rapid development of the economy and society, deep learning technology has increasingly attracted significant attention from both academia and industry due to its powerful data processing capabilities and broad application prospects. Convolutional Neural Networks have demonstrated excellent performance in tasks such as image recognition, object detection, and semantic segmentation. Meanwhile, the application of advanced technologies, including attention mechanisms, residual connections, and multi-scale feature pyramids, has further enhanced algorithm performance and robustness. Deep learning-based object detection algorithms can be primarily categorized into two major types: one-stage and two-stage approaches. Two-stage algorithms, represented by the R-CNN series, first generate candidate regions and then perform classification and regression on each candidate region. These algorithms achieve high detection accuracy but operate at relatively slower speeds. In contrast, one-stage algorithms such as YOLO and SSD directly predict object categories and locations within the network, achieving end-to-end detection. Although their accuracy is slightly lower, they operate faster and are more suitable for real-time application scenarios. Zhu et al. proposed the C2DEM-YOLO deep learning model, which achieves 92.31% mean average precision in photovoltaic electroluminescence (EL) defect detection through innovative design of the C2Dense feature extraction module and EMA cross-spatial attention mechanism [17]. Lang et al. proposed an innovative photovoltaic EL defect detection algorithm based on YOLO architecture, achieving 77.9% mAP50 detection accuracy on the PVEL-AD dataset through integration of polarized self-attention mechanisms and CNN-Transformer hybrid modules, representing a significant improvement of 17.2% over baseline methods [18]. Ding et al. proposed a photovoltaic EL defect detection method based on improved YOLOv5, effectively addressing defect sample imbalance and scale variation issues through an innovative design of Focal-EIoU loss function and cascade detection networks [19].

The rapid development of unmanned aerial vehicle (UAV) technology and significant cost reduction in recent years have made UAV-based intelligent inspection technology for photovoltaic power plants a focal point of industry attention. Wang et al. proposed an improved YOLOX model called PV-YOLO, which adopts the PVTv2 backbone network and CBAM attention mechanism, optimizes label assignment strategies, and employs the SIoU loss function while designing lightweight models of different scales to accommodate diverse detection scenarios [20]. Hong et al. proposed the CEMP-YOLOv10n algorithm, which achieves 86.6% accuracy and 87.3% mAP in photovoltaic infrared detection through ABCG_Block optimization for feature extraction, ERepGFPN enhancement for feature fusion, PConv streamlining for detection heads, and MECA attention mechanism for improved recognition accuracy. Simultaneously, the model’s computational load is reduced to 4.7 GFLOPs, providing an effective solution for UAV edge deployment and intelligent photovoltaic operation and maintenance [21]. Bommes et al. developed a semi-automatic photovoltaic module detection system based on UAV thermal imaging, which is projected to detect 3.5–9 MWp capacity daily with theoretical gigawatt-scale expansion potential [22]. Di Tommaso et al. proposed a multi-stage automatic detection model based on YOLOv3, achieving rapid and accurate diagnosis of multiple defect types in photovoltaic systems. The system, through multi-scale feature optimization and adaptive algorithm design, is compatible with different application scenarios including rooftop and ground-mounted power plants, with its defect severity classification functionality significantly enhancing the targeting of preventive maintenance [23]. UAV inspection technology demonstrates significant technical advantages and application value. In terms of efficiency, UAVs can rapidly traverse large-area regions, completing comprehensive detection of a 300 MW photovoltaic power plant within 2–3 days, representing a 10–20-fold improvement in detection efficiency compared to manual inspection and substantially shortening inspection cycles. From a safety perspective, UAVs employ remote control methods, effectively avoiding safety risks associated with maintenance personnel’s direct contact with high-voltage equipment, providing safer and more reliable solutions for power plant operation and maintenance. Regarding detection accuracy, UAVs possess hovering capabilities, enabling close-range photography above modules to acquire high-resolution detection images, with detection accuracy significantly superior to traditional manual inspection methods.

In terms of sensor technology, visible light cameras and thermal infrared cameras are core equipment for UAV inspection. Li et al. developed an automated detection platform for photovoltaic systems based on UAVs, achieving efficient identification of typical defects such as dust accumulation through the integration of visible light image processing algorithms and multi-sensor integration technology [24]. Visible light cameras can capture appearance information of photovoltaic modules, effectively identifying appearance defects including glass breakage, frame deformation, contamination and obstruction, and junction box appearance abnormalities. Their imaging principle is based on electromagnetic wave reflection in the visible light spectrum, offering advantages of high resolution, rich color information, and low cost. Thermal infrared cameras, based on infrared radiation emitted by objects themselves, can detect temperature distribution on module surfaces, effectively identifying thermal anomaly faults such as hot spots, cell cracks, junction box overheating, and local shading. Jamuna et al. proposed a linear iterative fault diagnosis system based on infrared thermal imaging, achieving precise detection and power optimization of photovoltaic module faults through the integration of thermal feature analysis and maximum power point tracking algorithms [25]. Vergura et al. systematically analyzed the influence mechanisms of dynamic variations in key parameters such as emissivity and reflected temperature, addressing radiation measurement errors caused by relative position changes during UAV infrared detection of photovoltaic modules [26]. These two imaging technologies exhibit clear complementarity: visible light images possess advantages in spatial resolution and detail representation, but cannot detect internal thermal anomalies; thermal infrared images can reveal invisible thermal faults but have relatively low spatial resolution and lack textural detail information. Winkel et al. proposed an electrothermal coupling analysis model that achieves a precise distinction between genuine defects and contamination artifacts in photovoltaic modules through the integration of spatial dirt distribution data from RGB images with temperature fields from infrared thermal imaging [27].

The introduction of dual-modal image fusion technology has provided new solutions for photovoltaic fault detection. Cardoso et al. proposed an intelligent monitoring system for photovoltaic modules based on UAV multimodal 3D modeling, achieving 99.12% accuracy improvement in module-level thermal characteristic analysis through the integration of RGB-thermal imaging photogrammetry technology [28]. Feng et al. proposed an intelligent detection framework for photovoltaic modules that integrates dual infrared cameras with deep learning, achieving high-speed module segmentation at 36 FPS through YOLOv5 and fault classification with 95% accuracy through ResNet, innovatively addressing the detection challenges of minute thermal defects in low-altitude environments [29]. Through simultaneous acquisition of visible light and thermal infrared images, combined with advanced image processing and artificial intelligence algorithms for dual-modal information fusion analysis, real-time online identification of typical defects such as cracks and hot spots has been achieved, enabling comprehensive and accurate detection of photovoltaic module faults [30]. Kuo et al. constructed an intelligent operation and maintenance system for photovoltaic power plants based on UAV multimodal image fusion, proposing a dual-channel CNN architecture that separately processes IR and RGB data to achieve high-accuracy comprehensive classification [31]. Dual-modal detection methods not only enable the detection of complex faults that cannot be identified by single-modal approaches but also improve detection robustness and reliability while reducing the influence of environmental factors on detection results.

Although the application of dual-modal image fusion technology in photovoltaic fault detection shows broad prospects, it still faces numerous complex technical challenges. Image registration constitutes a core challenge, as visible light and thermal infrared cameras exhibit significant differences in key parameters, including imaging principles, resolution, and field of view, necessitating the establishment of precise geometric correspondence relationships to ensure fusion accuracy. Ying et al. proposed an intelligent fault diagnosis scheme based on improved YOLOv5s and multi-level image registration, innovatively employing GPDF-AKAZE multi-scale registration technology to achieve 94.12% fault component localization recall rate in a 212.85 kW power plant [32]. Meanwhile, feature fusion presents equally critical challenges, requiring research on how to effectively integrate complementary information from two different modalities while fully utilizing their respective advantages, avoiding information redundancy and conflicts, and ensuring post-fusion information quality. Algorithm robustness represents another important challenge, as algorithms must adapt to image processing requirements under various complex conditions, including different illumination conditions, weather conditions, and shooting angles, considering the complex and variable operating environments of photovoltaic power plants. Centralized photovoltaic power plant component fault detection technology based on UAV dual-modal images represents an inevitable trend in photovoltaic industry technological advancement, possessing significant theoretical value and practical significance. This study aims to establish a multimodal photovoltaic component fault detection method suitable for UAV images, encompassing key components including dual-modal image acquisition, preprocessing, registration, fusion, and fault detection, providing technical support for intelligent operation and maintenance of photovoltaic power plants and promoting high-quality development of the photovoltaic industry. Through the organic combination of technological innovation and industrial application, this research expects to contribute scientific and technological strength to the global energy transition and carbon neutrality goals.

## 3. Materials and Methods

### 3.1. Data

#### 3.1.1. Data Acquisition

In photovoltaic module defect detection tasks, image data typically originates from two modalities: visible light and infrared. Visible light images possess high spatial resolution and rich textural details. The visible light imaging principle is primarily based on surface reflection characteristics, with imaging intensity (as shown in Equation (1)). The imaging intensity is directly proportional to external light sources; however, its performance is susceptible to interference from factors such as illumination variations, shadow occlusion, and specular reflection. In contrast, infrared images can reveal thermal anomaly characteristics within modules. The infrared imaging principle is based on thermal radiation emitted by objects themselves, with radiation intensity (as shown in Equation (2)). However, due to the imaging principle, infrared images often suffer from issues including blurred edges, weakened details, and sparse semantic information.

(1) $$I_{\mathrm{vis}}=\rho \left(\lambda \right)\times L_{\mathrm{incident}}\times \cos(\theta )$$

where I_vis_ denotes the intensity of visible light imaging, *ρ*(λ) represents the reflectance of an object at wavelength λ, L_incident_ indicates the intensity of incident light, and θ denotes the angle of incidence.

(2) $$I_{\mathrm{ir}}=\varepsilon \times \sigma \times T^{4}$$

where I_ir_ is the infrared radiation intensity, ε is the emissivity (0 < ε < 1), σ is the Stefan-Boltzmann constant (5.67 × 10^−8^ W/m^2^K^4^), and T is the absolute temperature of the object.

Due to the fundamental differences in the physical mechanisms of the two imaging modalities, they possess significant complementarity in information representation. Visible light images, by capturing reflected light information from object surfaces, retain rich high-frequency components in the frequency domain. Their Fourier transform amplitude spectrum exhibits a relatively slow decay (as shown in Equations (3) and (4)), thus clearly presenting surface morphological defects such as edges, cracks, and stains. In contrast, thermal infrared images, based on the thermal radiation characteristics of objects, have spectral energy concentrated in the low-frequency band, with rapid amplitude spectrum attenuation (as shown in Equations (5) and (6)). Although spatial details are more blurred, they are highly sensitive to temperature anomalies and can effectively identify functional defects such as hot spots and thermal breakdown. The two modalities provide complementary characterizations of photovoltaic module defects from geometric and thermodynamic dimensions, respectively.

(3) $$H_{vis}(u,v)=\iint I_{vis}(x,y)\times e^{-j2\pi (ux+vy)}dxdy$$

(4) $$|H_{vis}(u,v)|\propto \frac{1}{\sqrt{u^{2}+v^{2}}}$$

(5) $$H_{ir}(u,v)=\iint T(x,y)\times e^{-j2\pi (ux+vy)}dxdy$$

(6) $$|H_{ir}(u,v)|\propto \frac{1}{u^{2}+v^{2}}$$

where I_vis_(x,y) denotes the intensity of reflected light, and T(x,y) denotes the intensity of thermal radiation.

Based on the analysis of complementary characteristics in dual-modal imaging, constructing a high-quality multimodal photovoltaic fault detection dataset holds significant importance. However, due to the sporadic nature of photovoltaic faults and the strict control of operational data by photovoltaic enterprises for data privacy protection, acquiring high-quality photovoltaic fault samples—particularly multimodal visual images of photovoltaic panels—is extremely challenging. Not only is the collection process costly, but the data acquisition cycle is also protracted. A comprehensive review of existing public datasets revealed a lack of standardized datasets meeting the requirements of this study. Given this situation, this research employs unmanned aerial vehicle (UAV) inspection technology for data collection. The DJI M300 RTK industrial-grade quadcopter drone (DJI Innovation Technology Co., Ltd., Shenzhen, China) was selected as the data collection platform, equipped with the Zenmuse H20T hybrid sensor gimbal camera (DJI Innovation Technology Co., Ltd., Shenzhen, China). This camera integrates multiple sensor modules: a 20-megapixel 1/1.7-inch CMOS visible light camera captures high-definition visible light images at 5184 × 3888 pixels; The infrared sensor operates at 640 × 512 pixels, enabling precise detection of thermal anomalies in photovoltaic modules. The gimbal camera system features three-axis mechanical stabilization with a pitch axis range of −120° to +30°, maintaining image stability during flight and eliminating blur caused by drone attitude changes. The system supports simultaneous dual-modality imaging of visible light and thermal infrared with millisecond-level temporal synchronization, ensuring strict spatio-temporal correlation between multi-modal data. Based on these technical specifications, this study conducted systematic inspection imaging at multiple centralized photovoltaic power plants. Flight altitude was set between 20 and 30 m to ensure sufficient image resolution while maintaining safe clearance. This configuration enables simultaneous acquisition of high-resolution visible light and precise thermal infrared imagery, providing a high-quality data foundation for developing multimodal PV fault detection algorithms.

**Figure 1:**
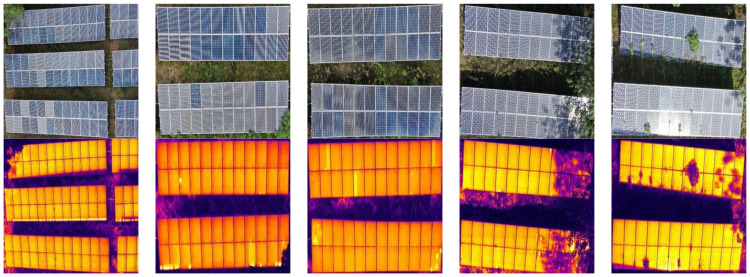
Fault Images of Components in Centralized Photovoltaic Power Stations.

During data preprocessing, static images were extracted from inspection videos using fixed-interval frame extraction. Rigorous image quality assessment criteria were then applied to systematically exclude low-quality images with redundant content or blurred targets. After meticulous screening, 1405 valid visible light images and their corresponding thermal infrared counterparts were obtained. All image pairs were captured simultaneously at identical times and perspectives, ensuring spatio-temporal registration accuracy for the multimodal data. Considering computational hardware constraints, particularly GPU memory limitations, the original high-resolution visible light images (5184 × 3888 pixels) could not be directly used for deep learning model training and inference. Additionally, to ensure consistent processing of multimodal data, the dimensions of visible light and thermal infrared images needed standardization. This study performed a series of spatial dimension normalization operations on the collected image data. Visible light images were downsampled to 640 × 640 pixels, reducing their field of view. Correspondingly, thermal infrared images underwent field-of-view cropping to achieve the same 640 × 640-pixel resolution. The processed fault images of centralized photovoltaic power station modules are shown in Figure 1.

#### 3.1.2. Dataset Creation and Preprocessing

Based on typical failure modes and sample distribution in photovoltaic systems, this study identifies three primary detection categories: hot spots, diode short circuits, and shading. These three failure types occur frequently in actual PV power plant operations and exhibit distinct thermal infrared characteristics. Hot spots typically manifest as localized high-temperature areas on the module surface. Diode short circuits generate abnormal heat accumulation at bypass diode locations, while shading faults appear as temperature differences between shaded and unaffected regions. Other fault types, such as module contamination, open circuits, and module displacement, also occur in PV systems. However, due to the relatively small sample sizes collected during data acquisition, these faults did not meet the statistical requirements for independent classification training. Effective training of deep learning models typically necessitates sufficient sample numbers per category to ensure model generalization and detection accuracy. Therefore, these fault types were not designated as separate detection categories. Annotation work was performed using the LabelImg (version 1.8.6) tool for precise labeling. This tool features an intuitive graphical interface and accurate bounding box drawing capabilities. The generated txt format label files.

**Figure 2:**
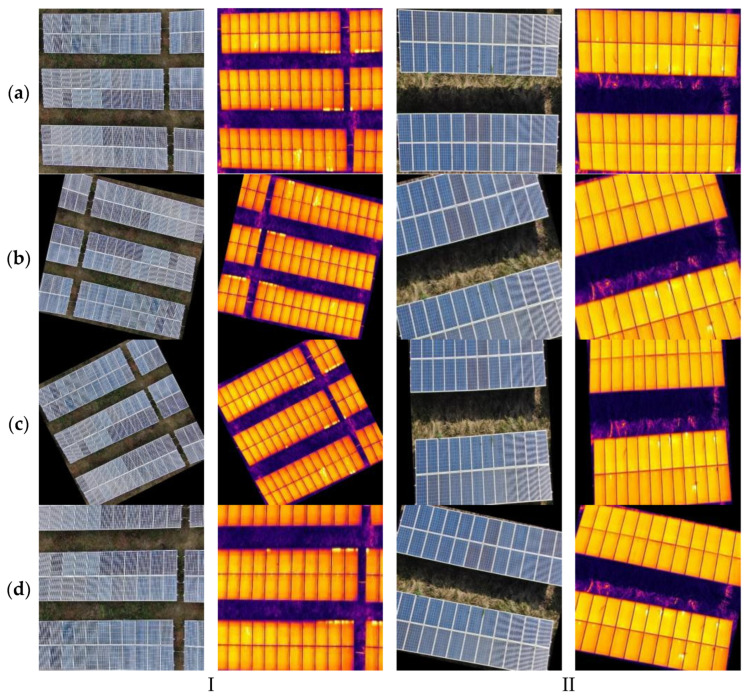
Image Enhancement. (**a**) Original image. (**b**–**d**) Dual-modality synchronized data-enhanced images. I and II are different examples.

The dataset was randomly split in an 8:1:1 ratio to form the training, validation, and test sets [33]. The construction of this dataset provides crucial data support for research on intelligent PV fault detection algorithms, effectively filling the gap in multimodal datasets within this field. To enhance the model’s generalization capability and detection performance, this study employs data augmentation strategies [34]. Addressing the requirement to process both visible light and thermal infrared images simultaneously for PV fault detection, a method was designed to ensure consistent spatial correspondence between the two modalities during augmentation. The specific augmentation strategies include horizontal flipping, vertical flipping, affine transformations, and random cropping. During experimentation, random augmentation operations were first applied to visible light images while recording transformation parameters. These identical parameters were then applied to corresponding thermal infrared images to guarantee precise spatial correspondence of target objects across both modalities. During data augmentation, bounding box coordinate transformations were automatically processed using YOLO-format normalized coordinate representation. Enhanced annotations were effectively filtered by setting a minimum visibility threshold of 0.1, expanding the dataset to four times its original size. The resulting training, validation, and test sets comprise 4500, 560, and 560 images, respectively, effectively mitigating the shortage of PV fault samples. This dataset is named RGBIRPV. Data augmentation is illustrated in Figure 2. This enhancement strategy provides the YOLO model with richer and more diverse training samples, enhancing its robustness and generalization capabilities in practical applications.

### 3.2. Basic YOLOv11 Model

This study is based on the YOLOv11 object detection framework [35]. While preserving the efficiency of single-stage detectors, YOLOv11 significantly improves model performance through systematic architectural optimizations. The algorithm retains the classic three-module design architecture comprising a backbone network, neck network, and detection head. Compared to YOLOv8, YOLOv11 introduces multiple innovative improvements across these modules. For the backbone network, this study employs a configurable C3k2 module as the core feature extractor. This module effectively improves the network’s adaptability to multi-scale features. The Neck integrates an innovative C2PSA module, which combines pointwise spatial attention mechanisms with enhanced feature expression through a multi-head attention architecture and feedforward neural networks. The detection head introduces two DWConv layers, substantially reducing computational complexity while maintaining detection accuracy, significantly improving model deployment efficiency. Collaborative optimization across modules enables YOLOv11 to deliver outstanding detection performance for multi-scale objects while preserving real-time detection capabilities. These enhancements make the model particularly suitable for industrial scenarios requiring real-time processing, such as photovoltaic panel defect detection. The model’s lightweight nature enables efficient operation on edge computing devices, meeting the stringent real-time detection requirements of intelligent PV plant operation and maintenance systems.

### 3.3. YOLOv11 Multimodal Model

During the operation of photovoltaic modules, common failure types include hot spots, diode short circuits, and shading. These faults are often difficult to detect comprehensively and accurately using a single-modality image. Visible light images provide an intuitive representation of the module surface’s structural morphology and external shading information. However, their ability to identify subtle defects is limited in complex environments such as low illumination or strong reflections. Infrared images, on the other hand, can precisely reflect abnormal temperature distributions within the module based on thermal radiation characteristics, making them a crucial tool for identifying hot spots and diode faults. Therefore, dual-modality image detection technology combining infrared and visible light has emerged as an effective solution to enhance the accuracy and robustness of photovoltaic fault identification.

In target detection, fusing visible and infrared images fully leverages the complementary spatio-temporal information of both modalities, endowing the fused image with more accurate and richer scene representation capabilities. Visible images, based on passive reflected light imaging, offer detailed richness but perform poorly under low-light or strong glare conditions. Infrared images, based on active thermal radiation imaging, exhibit strong anti-interference capabilities and effectively highlight target areas. Fusing these modalities balances image detail representation with target saliency, thereby enhancing detection model accuracy and robustness. By adapting the YOLOv11 object detection framework to a multimodal algorithmic structure, fusion strategies primarily fall into three categories: pixel-level fusion, feature-level fusion, and decision-level fusion [36].

**Figure 3:**
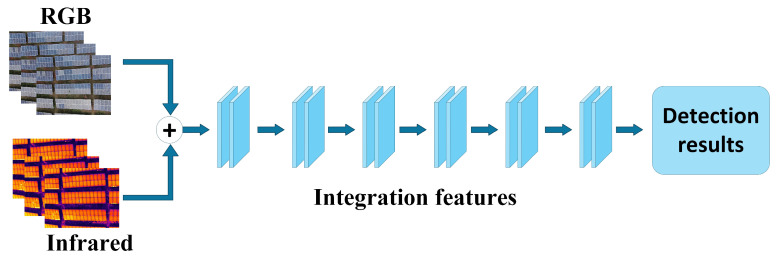
Pixel-level fusion.

Pixel-level fusion, also known as early fusion, involves merging multimodal images at the pixel level before data enters the deep learning network. This generates a single input image in a unified format, which is then fed into the object detection network for training and inference. Pixel-level fusion methods are illustrated in Figure 3. This approach requires no modification to traditional image processing network architectures. By performing simple pixel-level operations while preserving the original model structure, it achieves multimodal data fusion with advantages including ease of implementation, low computational complexity, and minimal resource overhead. In practical applications, pixel-level fusion is well-suited for scenarios demanding high real-time performance and low resource consumption. For instance, during photovoltaic module inspection, pixel-level fusion of visible light and infrared images simultaneously preserves structural texture information and temperature distribution features within a single image. This enhances the visibility and recognition accuracy of faulty areas. This approach effectively enhances the visual representation of various faults, such as hot spots and obstructions, providing robust support for improving the precision and automation of photovoltaic fault detection. It also lays the foundation for the broader adoption of multimodal information fusion technology in practical applications like photovoltaic operation and maintenance.

Feature-level fusion, also known as mid-level fusion, involves extracting feature representations from multi-modal input data through independent network branches. Subsequently, a dedicated fusion module is introduced at an intermediate network stage to integrate feature maps from different modalities, followed by joint feature extraction and task prediction. The core of this strategy lies in achieving cross-modal feature interaction and fusion at intermediate layers, thereby effectively capturing multi-source semantic information at varying depths and enhancing the richness and discriminative power of feature representations.

**Figure 4:**
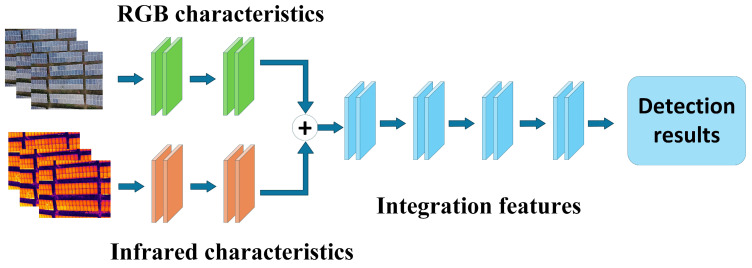
Feature-level early fusion.

**Figure 5:**
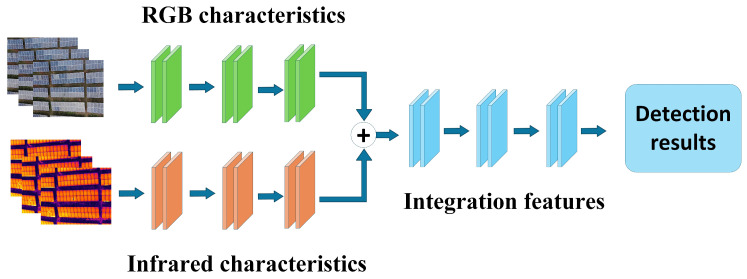
Feature-level mid-term fusion.

Based on the location of fusion, feature-level fusion can be further subdivided into early-stage feature fusion and mid-stage feature fusion. Early-stage feature fusion strategies integrate low-level features at shallow layers, as illustrated in Figure 4; mid-stage feature fusion integrates high-level semantic features at deep layers, as shown in Figure 5. Mid-level feature fusion strategies preserve the independent expressive capabilities of multimodal features while uncovering complementary relationships across modalities. This enables models to fully integrate multimodal information across multiple semantic levels, thereby enhancing perception capabilities and detection performance. Feature-level fusion offers high flexibility, allowing researchers to design diverse fusion mechanisms—such as feature concatenation, weighted summation, attention mechanisms, and cross-modal residual connections—based on specific task requirements and modal feature differences to achieve optimal fusion outcomes. Feature-level fusion offers high flexibility, enabling researchers to design diverse fusion mechanisms tailored to specific task requirements and modal feature differences. These include feature concatenation, weighted summation, attention mechanisms, and cross-modal residual connections, thereby achieving outstanding fusion performance.

**Figure 6:**
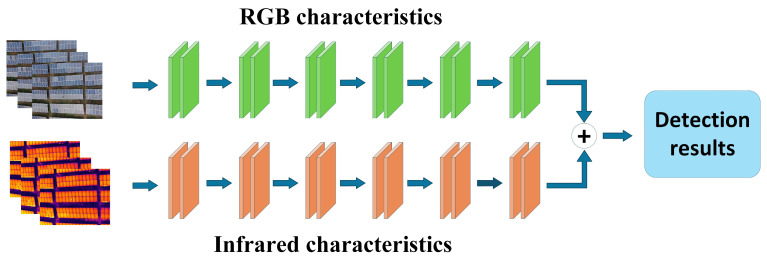
Decision-level integration.

Decision-level fusion, also known as post-fusion, refers to the process of integrating the final prediction results from each modality after independent feature extraction and object detection, enabling joint decision-making, as shown in Figure 6. Each modality outputs a series of candidate bounding boxes and corresponding confidence scores through its independent detector. The system then fuses these detection results according to a specific strategy during the output stage to derive the final detection conclusion. Common fusion strategies include confidence-weighted averaging, voting mechanisms, and intersection-over-union operations on bounding boxes. The core of these strategies lies in leveraging the complementary nature of independent detectors across modalities to enhance the system’s overall robustness and fault tolerance without altering the original network architecture. Decision-level fusion offers significant advantages in terms of high flexibility and ease of implementation. Modal detection models can adopt existing mature architectures and be deployed independently without sharing weights or feature representations. Only a lightweight result fusion module needs to be added at the end. This approach significantly improves system reliability. If one modality temporarily fails due to environmental factors, the system can still make effective judgments as long as other modalities retain detection capabilities.

### 3.4. RFE-YOLO Model

**Figure 7:**
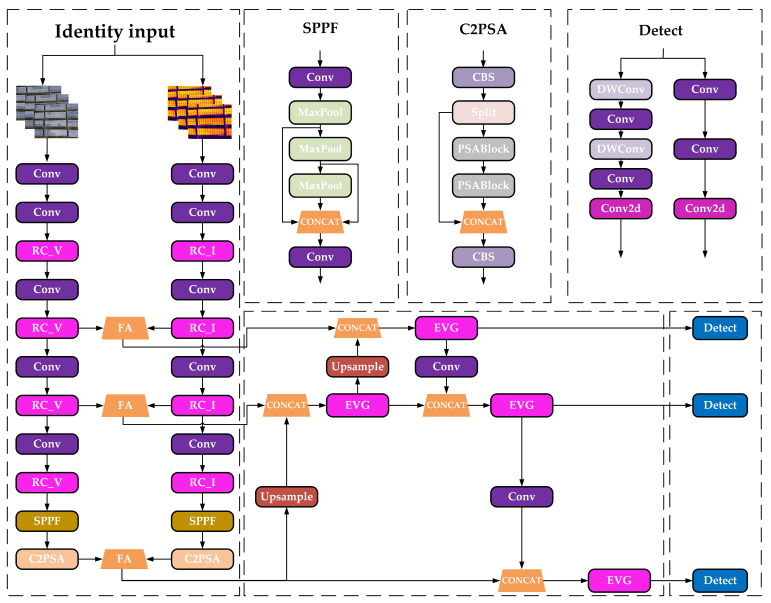
RFE-YOLO Model.

To achieve precise identification of photovoltaic module faults, this study selected feature mid-term fusion YOLOv11n as the foundational model for photovoltaic module fault detection. Addressing challenges encountered during photovoltaic module fault detection, this paper proposes the RFE-YOLO model. The RFE-YOLO photovoltaic module fault detection network architecture is illustrated in Figure 7.

The RFE-YOLOv11 Model leverages the complementary nature of visible light and infrared modalities by designing the RC module. This module constructs dedicated RepConv feature extraction units for each modality, integrating CBAM attention mechanisms with differentiated convolution kernel configurations to achieve adaptive enhancement of visible light detail features and infrared global thermal features. During feature fusion, the FA module introduces lightweight channel and spatial attention mechanisms alongside learnable modality balance factors to achieve efficient adaptive cross-modal feature integration. Concurrently, the EVG module employs deep separable convolutions to significantly reduce parameter counts while preserving local texture receptive fields. Combined with channel attention mechanisms, it enables modality-adaptive channel reweighting.

#### 3.4.1. Feature Extraction RC Module

This study constructs a multimodal RC reparameterized feature extraction architecture for visible-infrared images based on the CBAM attention mechanism. The core innovation lies in designing heterogeneous RC_V and RC_I feature extraction modules, enabling differentiated feature extraction and enhancement for visible and infrared modality data. CBAM addresses the limitations of traditional CNNs in capturing fine-grained spatial details and highlighting key feature channels [37]. When applied to photovoltaic panel feature extraction in visible and infrared images, CBAM enhances the model’s perception of critical areas such as hotspots and different spectral channels. This enables more effective multimodal information fusion and improves detection accuracy. CBAM comprises CAM and SAM: CAM models channel dependencies through fully connected layers to strengthen critical information channels; SAM extracts spatial responses via max pooling and average pooling to highlight prominent regions.

**Figure 8:**
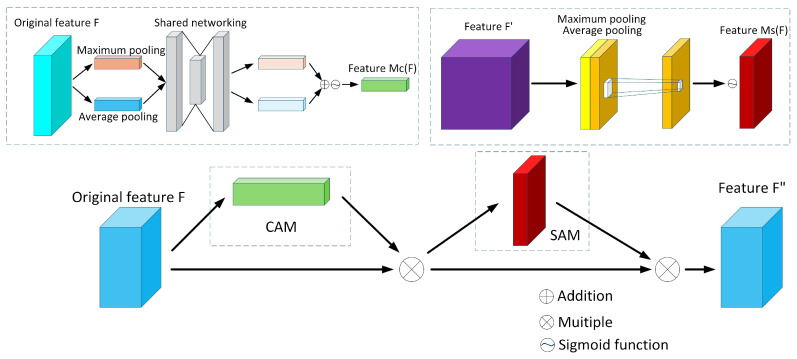
CBAM Attention Mechanism.

The CBAM framework, as illustrated in Figure 8, feeds its feature map into CAM. Through parallel global average pooling and max pooling operations, it extracts spatial context information, generating vectors $F_{\mathrm{avg}}^{C}$ and $F_{\max}^{C}$ that describe global features, respectively. These two vectors are modeled via an MLP with shared weights and fused through element-wise addition to produce channel attention weights, as shown in Equations (7)–(10).

(7) $$F_{\mathrm{avg}}^{C}=\frac{1}{H\times W}\sum_{i=1}^{H}\sum_{j=1}^{W}F_{i,j}$$

(8) $$F_{\max}^{C}=\max_{i=1}^{H}\max_{j=1}^{W}F_{i,j}$$

(9) $$\begin{array}{ll} M_{c}(F) & =\delta \left(\mathrm{MLP}(\mathrm{AvgPool}(F))+\mathrm{MLP}(\mathrm{MaxPool}(F))\right)\\ & =\delta \left(W_{1}(W_{0}(F_{\mathrm{Avg}}^{c}))+W_{1}(W_{0}(F_{\mathrm{Max}}^{c}))\right) \end{array}$$

(10) $$F^{\prime }=M_{c}(F)⊗F$$

SAM utilizes projections of average pooling and max pooling along the channel dimension to obtain two spatial mappings, A and B, representing the average response and maximum response at each location, respectively. After concatenating these two mappings, they are fed into a convolutional layer to generate a spatial attention map, as calculated by Equations (11)–(14).

(11) $$F_{avg}^{S}=AvgPool(F)=\frac{1}{C}\sum_{c=1}^{C}F^{\prime i}$$

(12) $$F_{max}^{S}=MaxPool(F)=max_{i=1}^{C}F^{\prime i}$$

(13) $$\begin{array}{ll} M_{s}(F) & =\delta \left(f^{7\times 7}\left[AvgPool(F);MaxPool(F)\right]\right)\\ & =\delta \left(f^{7\times 7}\left(\left[F_{Avg}^{s};F_{Max}^{s}\right]\right)\right) \end{array}$$

(14) $$F^{\prime \prime }=M_{s}(F^{\prime })⊗F^{\prime }$$

**Figure 9:**
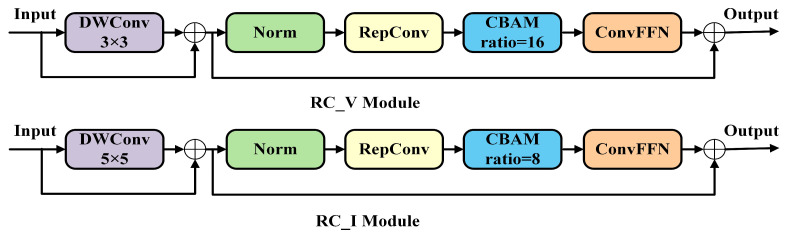
RC Module.

The proposed RC module is illustrated in Figure 9. The network first preprocesses the input image using residual deep convolutions: within the visible light branch RC_V module, residual connections via 3 × 3 deep convolutions preserve local texture integrity. Following layer normalization and reparameterization processing [38], combined with a CBAM attention mechanism was used with a compression ratio of 16 to enhance edge feature responses. Adaptive detail variation is achieved through the multiscale design of deformable convolution blocks [39]. In the infrared branch RC_I module, a 5 × 5 large-kernel deep convolution with residual connections expands the receptive field to capture global distribution patterns of thermal radiation. This is combined with a CBAM attention mechanism with a compression ratio of 8 to preserve thermally sensitive channel information, while a hierarchical convolutional structure models the global spatial correlations of thermal targets. Both branches ultimately employ a ConvFFN for feature regularization and distribution stabilization [40], with residual connections fusing the output features. This RC architecture, based on CBAM attention, combines channel-spatial joint attention with modality-customized feature enhancement strategies, enabling differentiated feature representation capabilities for visible and infrared images.

#### 3.4.2. FA Module

**Figure 10:**
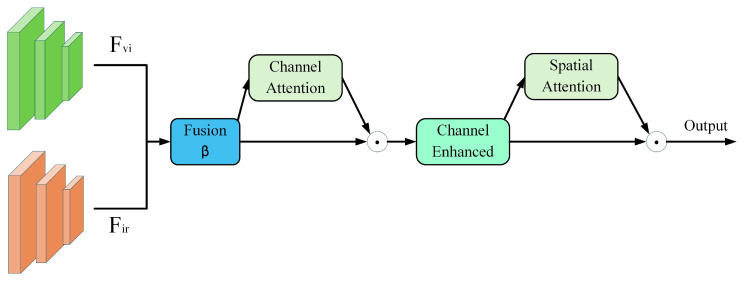
FA Module.

In the task of fusion detection using infrared and visible light images for photovoltaic panels, the two modalities exhibit significant complementary characteristics. Infrared images effectively capture thermal distribution anomalies such as hot spots and localized overheating defects, while visible light images excel at characterizing structural information, facilitating the identification of surface defects like cracks, obstructions, and contamination. However, in practical applications, the quality of information from these two modalities varies significantly under different environmental conditions: visible light image quality deteriorates sharply under strong illumination or reflective interference; infrared images suffer from insufficient contrast in low-temperature-difference environments. Therefore, designing a mechanism that can adaptively fuse bimodal information and dynamically enhance key features becomes crucial for improving detection performance. To address these challenges, this paper proposes the FA module, as illustrated in Figure 10. This module achieves efficient feature fusion through a lightweight design, employing a learnable modality balance strategy and a cascaded attention mechanism. This approach significantly enhances feature expression capabilities while maintaining computational efficiency.

The FA module first achieves dynamic fusion of dual-modal features through an adaptive modal balancer. This mechanism introduces a learnable parameter β to control the weight distribution between the two modalities, as shown in Equation (15), initialized at 0.5 to ensure balance during the early training phase.

(15) $$F_{base}=\beta \times F_{vi}+(1-\beta )\times F_{ir}$$

$F_{vi}\in R^{B\times C\times H\times W}$ and $F_{ir}\in R^{B\times C\times H\times W}$ represent visible light and infrared features, respectively. Unlike fixed-weight fusion methods, the parameter β is automatically adjusted during training based on gradient feedback, enabling the model to optimize modal weight allocation according to varying scene conditions and fault types. This design is particularly well-suited for photovoltaic inspection scenarios where modal signal-to-noise ratios undergo dynamic changes.

To enhance the expressive capability of fusion features in the semantic dimension, the FA module designs a channel attention mechanism based on a compression-excitation strategy. This mechanism employs global adaptive average pooling to capture global contextual information and implements nonlinear feature transformation through a lightweight network composed of two pointwise convolution layers, as shown in Equations (16)–(19).

(16) $$A_{c}=\sigma (W_{2}\times ReLU(W_{1}\times GAP(F_{base})+b_{1})+b_{2})$$

(17) $$W_{1}\in R^{C_{hidden}\times C}$$

(18) $$W_{2}\in R^{C\times C_{hidden}}$$

(19) $$C_{hidden}=max(C/r,4)$$

This study sets the compression ratio to r = 64, aiming to reduce the number of parameters while maintaining sufficient expressive power. Channel enhancement feature calculation is shown in Equation (20).

(20) $$F_{channel}=F_{base}⊙A_{c}$$

By recalibrating the importance weights of each channel, this mechanism highlights semantic features highly correlated with PV faults while effectively suppressing background noise and redundant information. Building upon channel enhancement, the FA module further introduces a spatial attention mechanism to strengthen the model’s perception of critical regions. This mechanism extracts discriminative information from the spatial dimension through statistical pooling operations. After concatenating the two feature maps, spatial attention weights are generated via 3 × 3 convolutions, as shown in Equations (21)–(23).

(21) $$F_{avg}=\frac{1}{C}\sum_{i=1}^{C}F_{channel}^{\left(i\right)}$$

(22) $$F_{max}=max_{i=1}^{C}F_{channel}^{\left(i\right)}$$

(23) $$A_{s}=\sigma (Conv_{3\times 3}(Concat(F_{avg},F_{max})))$$

The final output features are shown in Equation (24).

(24) $$F_{out}=F_{channel}⊙A_{s}$$

This design enables the model to adaptively focus on potential fault regions in spatial dimensions, such as hot spots or obscured areas, thereby enhancing the precision of fault localization. The FA module achieves deep fusion and precise enhancement of bimodal information while maintaining computational efficiency through the organic integration of an adaptive modal balance mechanism and a cascaded attention enhancement strategy. Learnable modal balance parameters enable the model to dynamically adjust weight distribution based on varying environmental conditions and fault types, effectively addressing the challenge of dynamic modal signal-to-noise ratio fluctuations in PV detection. The lightweight channel attention mechanism achieves a 64:1 parameter compression ratio, significantly reducing computational overhead. Meanwhile, the spatial attention mechanism enhances perception of critical regions through statistical pooling strategies. The cascaded dual-attention design refines feature fusion across semantic and spatial dimensions, ensuring stable and accurate detection performance even under strong reflective illumination and background interference. This provides more discriminative feature representations for subsequent processing.

#### 3.4.3. EVG Module

To address the challenges of uneven distribution and strong complementary information in multimodal image features, this paper introduces the EVG module, inspired by the GSConv architecture, to enhance the efficiency of shallow feature extraction and the expressive power of deep semantic fusion. The GSConv module demonstrates significant advantages in feature compression and local modeling. By integrating lightweight convolutions with a channel reordering mechanism, it effectively captures subtle yet critical texture structures and thermal anomaly edge information in infrared images while maintaining computational efficiency. This capability demonstrates excellent responsiveness to fine-grained defect targets commonly found in infrared images. Furthermore, the EVG module enhances the model’s fusion capability for multi-scale semantic features during the neck stage by constructing a multi-layer efficient bottleneck structure and introducing a channel attention mechanism. Its design not only achieves information interaction and retention between main and branch paths but also strengthens the model’s focus intensity on minute fault regions through the channel attention mechanism.

**Figure 11:**
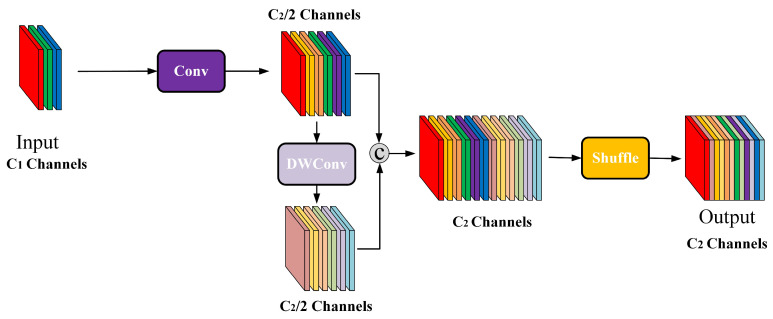
GSConv Module.

The GSConv module is illustrated in Figure 11. One path employs standard convolutions to compress input channels and perform preliminary linear transformations; The second path applies depthwise separable convolutions to the compressed feature map, enhancing the receptive field while reducing coupling in cross-channel computations. The outputs from both subpaths are concatenated and then shuffled through a channel-rearrangement mechanism to boost feature diversity and expressiveness, as illustrated in Equations (25)–(27). Compared to traditional convolutions, GSConv significantly reduces computational load while maintaining the same number of output channels. Consequently, in infrared images where target boundaries are blurred and texture features are weak, its large receptive field and sparse connections help strengthen feature perception in low-contrast regions.

(25) $$X_{1}=\sigma (W⊗X)$$

(26) $$X_{2}=\sigma (BN(W_{dw}⊗X_{1}))$$

(27) $$X_{3}=Shuffle([X_{1},X_{2}])$$

**Figure 12:**
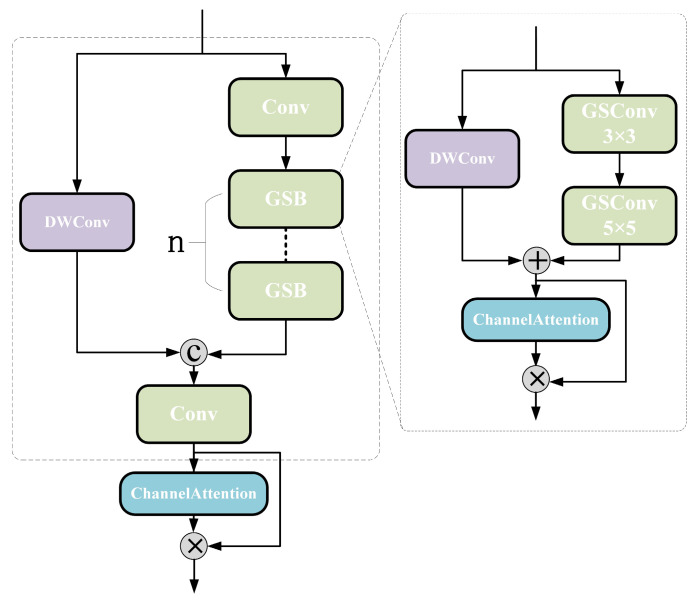
EVG Module.

The EVG module, as shown in Figure 12, adopts a dual-path parallel architecture, enhancing gradient flow and information transfer efficiency through feature reuse mechanisms. Input features are processed through two parallel subpaths: the main path first undergoes channel dimension reduction via standard convolutions, then feeds into multiple cascaded GSB modules for deep semantic feature extraction; the auxiliary path employs deep separable convolutions to construct feature-preserving connections, efficiently preserving original spatial details and low-level feature information.

The GSB module consists of two cascaded GSConv units, enabling comprehensive extraction of fine-grained spatial and channel features while maintaining low computational complexity, as shown in Equation (28). During feature fusion, this module introduces a channel attention mechanism. It captures channel-level statistical features by jointly employing global average pooling and max pooling, followed by cross-channel interaction and nonlinear fusion through a shared multilayer perceptron. The generated attention weights undergo normalization before dynamically acting on the fused features, enabling targeted enhancement of key channels and effective suppression of redundant information. The EVG module concatenates the output features from both paths along the channel dimension, performs cross-channel information fusion via pointwise convolution, and utilizes the channel attention mechanism to adaptively weight and optimize the output features. This further enhances the model’s feature discrimination capability and computational efficiency, as shown in Equations (29) and (30).

(28) $$F_{fusion}=DWConv(X_{input/2})\|Conv_{1\times 1}\left(GSB\left(Conv(X_{input})\right)\|DWConv(X_{input/2})\right)$$

(29) $$M_{c}=\sigma \left(FC\left(AvgPool(X_{fusion})\right)+FC\left(MaxPool(X_{fusion})\right)\right)$$

(30) $$F_{\mathrm{out}}=F_{\mathrm{fusion}}⊗M_{c}$$

## 4. Results

### 4.1. Experimental Environment and Parameter Settings

All experiments in this study were conducted under a unified hardware and software environment to ensure the reliability and reproducibility of results. Python 3.8 was used as the programming language, with model training and testing performed on the PyTorch 2.2.0 deep learning framework. The hardware configuration utilized an NVIDIA RTX 4090 GPU (NVIDIA Corporation Santa Clara, CA, USA) equipped with 24GB of VRAM, providing ample computational resources for model training.

Detailed experimental environment configuration information is shown in Table 1. Key model training parameter settings are listed in Table 2. These detailed environmental and parameter configurations ensure experimental transparency, providing a reliable foundation for reproducing and comparing subsequent research.

### 4.2. Model Evaluation Metrics

To comprehensively evaluate the performance of the constructed multimodal PV fault detection model, this paper employs multiple evaluation metrics commonly used in the object detection field, including precision, recall, average precision, and mean average precision. These metrics are calculated based on the statistical results of the four fundamental elements in the confusion matrix: T_P_, F_P_, T_N_ and F_N_. Different metrics reflect the model’s detection capabilities from distinct dimensions, holding significant importance in photovoltaic module fault detection.

Precision represents the proportion of samples that are actually faulty among all samples predicted as faulty by the model. This metric measures the model’s ability to control the misclassification of normal components as faulty, which helps reduce unnecessary maintenance costs in practical engineering applications. The calculation formula is:

(31) $$P=\frac{T_{P}}{T_{P}+F_{P}}$$

Recall measures the proportion of all actual defect samples that the model correctly identifies. It reflects the model’s ability to detect defects, with higher recall indicating fewer missed defects. This is particularly significant in early defect detection and safety monitoring. The calculation formula is:

(32) $$R=\frac{T_{P}}{T_{P}+F_{N}}$$

Since precision and recall often involve a trade-off relationship, the F1 score is introduced as a comprehensive evaluation metric to assess overall model performance. The calculation formula is:

(33) $$F_{1}=\frac{2PR}{P+R+\xi }$$

Here, ξ is a minimal constant that prevents the denominator from becoming zero.

Average Precision reflects a model’s detection accuracy at different confidence thresholds by calculating the area under the precision-recall curve for a given class. Specifically, AP integrates or averages the precision values corresponding to different recall points between 0 and 1 for a target class, thereby providing a comprehensive evaluation of the model’s overall performance on that class. The formula is:

(34) $$AP=\int_{0}^{1}P(R)dR$$

The mean average precision (mAP) represents the average precision of all categories, reflecting the model’s overall detection accuracy across the entire dataset. The calculation of mAP relies on the intersection-over-union (IoU) threshold between predicted results and ground truth bounding boxes. The mAP metric carries different evaluative significance under varying threshold conditions. The formula is:

(35) $$mAP=\sum_{i=1}^{M}\frac{AP}{M}$$

### 4.3. Multi-Modal Fusion YOLOv11 Model

To comprehensively evaluate the performance of different fusion strategies in multimodal object detection tasks, this study systematically compares the detection performance of four mainstream fusion strategies based on the RGBIRPV dataset using the YOLOv11 multimodal fusion network architecture. Specifically, the pixel-level fusion strategy directly concatenates RGB and infrared image data at the input layer. While it preserves complete raw information and spatial details, it significantly increases the input dimensionality and computational complexity of the network and may introduce redundant information. The early feature-level fusion strategy integrates multimodal features during the shallow feature extraction stage of the network, enabling full utilization of complementary information between modalities and establishing effective cross-modal feature associations in the early stages of network training. The mid-level feature fusion strategy performs feature integration in the intermediate layers of the network, achieving a favorable balance between feature representation capability and computational efficiency. The decision-level fusion strategy combines the prediction results of each modal branch at the output layer, which, while maintaining the independence of each modality, exhibits a relatively shallow degree of fusion.

Experimental results demonstrate that the mid-level feature fusion strategy achieves the best performance in terms of mAP@50 detection accuracy (as shown in Table 3). By performing deep fusion at the intermediate stage of multimodal feature extraction, this strategy achieves an optimal balance between computational cost and model performance, effectively integrating complementary information from different modalities and enabling the network to establish robust cross-modal associations at critical stages of feature learning.

### 4.4. Cross-Validation Experiments

To evaluate the model’s robustness and generalization capability, this study employs a five-fold cross-validation method. The training and validation sets are combined into a dataset comprising 5060 images, which is uniformly divided into five subsets. In each round of experimentation, four subsets are used for training while the remaining subset serves as validation. This process is repeated five times, ensuring each subset is used for validation once. This approach reduces bias introduced by data partitioning and enhances the reliability of evaluation results.

Each round of experiments followed a standard training procedure, with performance metrics—including precision, recall, F1 score, and mean average precision (mAP)—calculated on the validation set. Upon completing all experiments, results from each round were aggregated to compute the mean and standard deviation of metrics, enabling a robust estimation of model performance. Experimental results are presented in Table 4.

As shown in Table 4, the model demonstrates overall stable performance across five-fold cross-validation. The average mAP@50 reaches 82.3% with a standard deviation of only 1%, indicating minimal fluctuation across different datasets and strong robustness. The standard deviations for Precision and Recall are 1.5% and 2.1%, respectively, suggesting consistent performance across datasets. Concurrently, the standard deviation of the F1 score is only 0.8%, further validating the model’s balanced trade-off between precision and recall. Based on the cross-validation results, the model with the optimal mAP performance on the validation set was ultimately selected for evaluation on the test set. Table 5 presents a comparison between this model’s best results on the validation set and its performance on the test set.

As shown in Table 5, the model with the best performance on the validation set exhibits slight fluctuations in overall performance on the test set. mAP@50 decreased from 83.4% to 83.2%, F1 score dropped from 79.7% to 78.3%, and Precision fell from 83.5% to 80.1%. Despite these declines, the small magnitude indicates the model maintains stable generalization performance on unseen data. Overall results demonstrate that the model retains high detection accuracy even in complex scenarios.

### 4.5. Ablation Experiment

To validate the effectiveness and practicality of an improved fault detection scheme for multimodal photovoltaic modules, this paper proposes a feature-level mid-term fusion detection method combining visible and infrared images based on the YOLOv11n baseline model. Experiments were conducted using a self-built RGBIRPV dataset. Under identical experimental settings, the specific contributions of each module to model performance were quantified. Evaluation metrics encompass detection accuracy and model complexity dimensions, including precision, recall, mAP@50, mAP@50:95, F1 score, number of parameters, floating-point operations, and model volume.

#### 4.5.1. RC Module Experiment

To investigate the impact of the attention compression ratio on feature extraction across different modalities, we conducted ablation studies on the RC_V and RC_I modules. As summarized in Table 6, the configuration with RC_V = 16 and RC_I = 8 enabled the model to most effectively leverage the complementary properties of bimodal features: the visible branch excelled at preserving fine details and textures, while the infrared branch showed enhanced sensitivity in thermal feature extraction and anomaly localization. Compared to a uniform compression ratio, this asymmetric design significantly improved detection performance, achieving a precision of 83.8%, a recall of 74.1%, and an mAP@50 of 83.0%. These results collectively validate the rationality and effectiveness of the proposed modality-specific attention differentiation strategy.

This study systematically evaluated the impact of heterogeneous convolutional kernel parameters on multimodal feature extraction in the RC_V and RC_I modules via ablation experiments. As presented in Table 7, the model performed best with the RC_V = 3 and RC_I = 5 configuration, with a precision of 83.8%, mAP@50 of 83.0%, and F1-score of 78.6%. This outcome indicates that this kernel combination significantly boosts detection accuracy and mean average precision, validating that using small kernels for detail in the visible branch and large kernels for global context in the infrared branch is an effective strategy.

#### 4.5.2. Module Ablation Experiment

Table 8 ablation experiments demonstrate that the YOLOv11n model integrated at the feature level achieves benchmark performance in photovoltaic module fault detection: *p* = 79.6%, R = 76.3%, mAP@50 = 81.9%, mAP@50:95 = 66.7%, F1 = 77.9%, with 3.95 parameters, 9.5 GFLOPs of floating-point operations, and a model size of 8.3 MB. These results demonstrate the baseline model’s capability to detect faulty modules, though it remains suboptimal in achieving high threshold accuracy and lightweight design. In single-module enhancements, introducing the RC module strengthened the model’s edge feature extraction for PV module defects, improving *p*-value by 4.2%, mAP@50 by 1.1%, and F1 score by 0.8%, while reducing parameters by 5.8%. However, R-value decreased by 2.2%, indicating that this architecture may sacrifice some false-negative detection control capabilities while enhancing localization accuracy. After introducing the FA module, the R value remained largely stable, while the F1 score improved to 78.7% and mAP@50 increased by 0.6%. This indicates that the feature aggregation mechanism enhances global information modeling capabilities, positively impacting multi-class defect detection. Adding the EVG module reduced parameters by 7.6% while maintaining mAP@50:95 = 67.1%. Despite minor fluctuations in accuracy metrics, its lightweight design enables feasible deployment on resource-constrained monitoring equipment in photovoltaic power plants. In the fusion approach, the RC and FA modules achieve a balance between performance and reliability. mAP@50 increases to 83.3%, and mAP@50:95 grows by 0.5%, while parameter size and GFLOPs consumption decrease. This demonstrates that heavy-parameter convolutions and feature aggregation mechanisms play a facilitating role in PV fault detection. The RC-EVG combination achieves mAP@50:95 = 79.3%, while increasing the R value by 1.1% and the F1 value by 1.4%, demonstrating strong recognition capabilities for localized micro-hotspot faults. However, GFLOPs increased to 10.1, leading to higher computational overhead. The FA-EVG model maintains detection stability while boosting the R value to 77.7% and reducing parameters to 3.59 million. With a model size of only 7.7 MB, it holds excellent prospects for edge deployment.

Ultimately, the proposed RFE-YOLO model achieved performance metrics of *p* = 82.5%, R = 76.5%, mAP@50 = 83.7%, mAP@50:95 = 67.6%, and F1 = 79.4%. Compared to the baseline model, this approach reduced the number of parameters by 6.6% while maintaining a model size below 8.0 MB. Experimental results demonstrate that RFE-YOLO maintains high detection robustness under multi-threshold conditions while balancing lightweight design with accuracy. This model is suitable for deployment on edge computing devices, enabling rapid and precise identification of various photovoltaic module fault types.

### 4.6. Comparison Experiments of Different Models

To evaluate the effectiveness of photovoltaic module fault detection and ensure a comprehensive comparison, the experimental results cover both single-modal and multi-modal scenarios. The comparison focuses on multiple metrics, including *p*, R, mAP, F1, parameter, GFLOPs, and model size.

Single-modal detection models based on infrared images include YOLOv5n, YOLOv6n, YOLOv8n, YOLOv9t, YOLOv10n, YOLOv11n, YOLOv12n [41], and RT-DETR-l [42]. Multi-modal detection models based on visible and infrared image fusion include mid-fusion-YOLOv11n, DEYOLO [36], ICAFusion [43], and RFE-YOLO. This table enables a comprehensive evaluation of the performance differences among the proposed methods. Specific performance metric comparisons are shown in Table 9.

In single-modal infrared image experiments, YOLOv11n demonstrated superior performance in photovoltaic module fault detection tasks. Specifically, this model achieved the best results across three core metrics: mAP@50 (80.6%), mAP@50:95 (60.8%), and F1 score (77.0%). It also attained a recall rate of 77.1%, the highest among all compared models, demonstrating strong fault detection capability and a low risk of missed detections. In contrast, while YOLOv12n holds an advantage in precision and RT-DETR-l excels in mAP@50:95, both suffer from substantial model size and computational overhead, making them unsuitable for deployment in resource-constrained real-world scenarios. YOLOv11n, however, achieves both high accuracy and efficiency with only 2.58 million parameters, 6.3 GFLOPs, and a model size of 5.5 MB, fully demonstrating its outstanding lightweight characteristics and practical value.

In multimodal visible and infrared image experiments, RFE-YOLO demonstrated the most outstanding overall performance. This model achieved the best results across four key metrics: precision of 82.5%, mAP@50 of 83.7%, mAP@50:95 of 67.6%, and F1 score of 79.4%. While achieving a recall rate of 76.5%. This indicates not only more accurate localization of PV module faults but also effective reduction in missed detection rates, ensuring the completeness and reliability of detection results. In contrast, feature-level mid-stage fusion models like YOLOv11n and ICAFusion exhibit insufficient generalization capabilities at high IoU thresholds. While DEYOLO demonstrates superior precision, its large parameter count and model size limit practical deployment. Notably, RFE-YOLO achieves high-precision detection in complex scenarios with a lightweight configuration requiring only 3.69 parameters and an 8.0 MB model size, further highlighting its practical value in PV plant operations and maintenance. Aligning with real-world application needs, RFE-YOLO advantages extend beyond significant improvements in experimental metrics to include deployability. For large-scale inspection tasks in photovoltaic power plants, detection models must simultaneously deliver high accuracy and efficiency. RFE-YOLO achieves rapid inference with low computational overhead while maintaining detection performance, making it highly suitable for deployment on drones or edge computing devices. This demonstrates that the method not only enhances fault detection accuracy but also significantly boosts its feasibility and application potential in real-world photovoltaic operations and maintenance environments.

**Figure 13:**
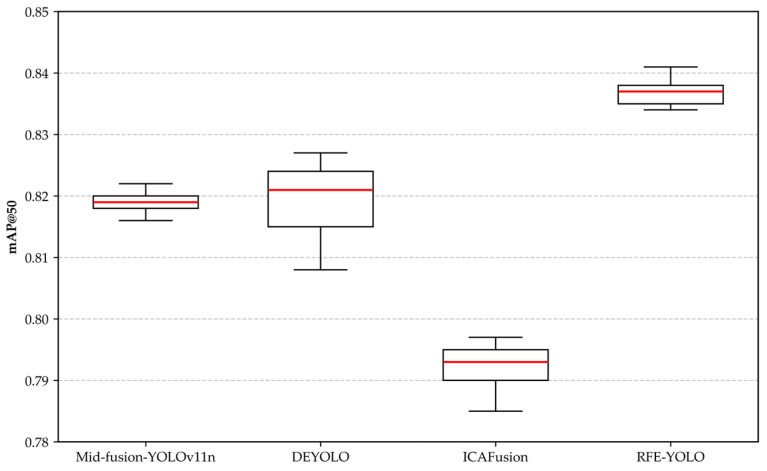
Box plot comparison of experimental results from different models.

In the RGB and IR dual-modality comparison experiments, four models were selected for comparison: mid-stage fusion YOLOv11n, DEYOLO, ICAFusion, and RFE-YOLO. Each set of experiments was independently repeated five times, and the results were plotted as box plots to demonstrate the performance distribution and stability of different models across multiple experiments, as shown in Figure 13. Given the high dependency of photovoltaic module fault detection tasks on detection accuracy, this study employs mAP@50 as the key comparison metric. This metric authentically reflects the stability of fault localization and accuracy in photovoltaic module fault detection, aligning closely with real-world requirements for stable, reliable detection results that minimize missed faults.

As shown in Figure 13, RFE-YOLO demonstrates significantly superior performance compared to other methods on the mAP@50 metric. Statistically, RFE-YOLO achieves an average score of 0.837, surpassing Mid-fusion-YOLOv11n, DEYOLO, and ICAFusion by 2.2%, 2.2%, and 5.7%, respectively. The box plot reveals that the performance distribution range of RFE-YOLO shows almost no overlap with other methods, indicating its significant performance advantage. Particularly compared to ICAFusion, RFE-YOLO demonstrates a notable improvement in detection accuracy. Notably, RFE-YOLO not only excels in average performance but also surpasses the average minimum values of Mid-fusion-YOLOv11n and DEYOLO, fully demonstrating the method’s robustness and stability. Although DEYOLO exhibits an upward performance trend in consecutive experiments, RFE-YOLO achieves a superior balance between detection accuracy and model stability through its effective integration of multi-scale feature extraction fusion mechanisms. This makes it a more reliable and efficient solution for object detection tasks in complex environments.

### 4.7. Visual Analytics

**Figure 14:**
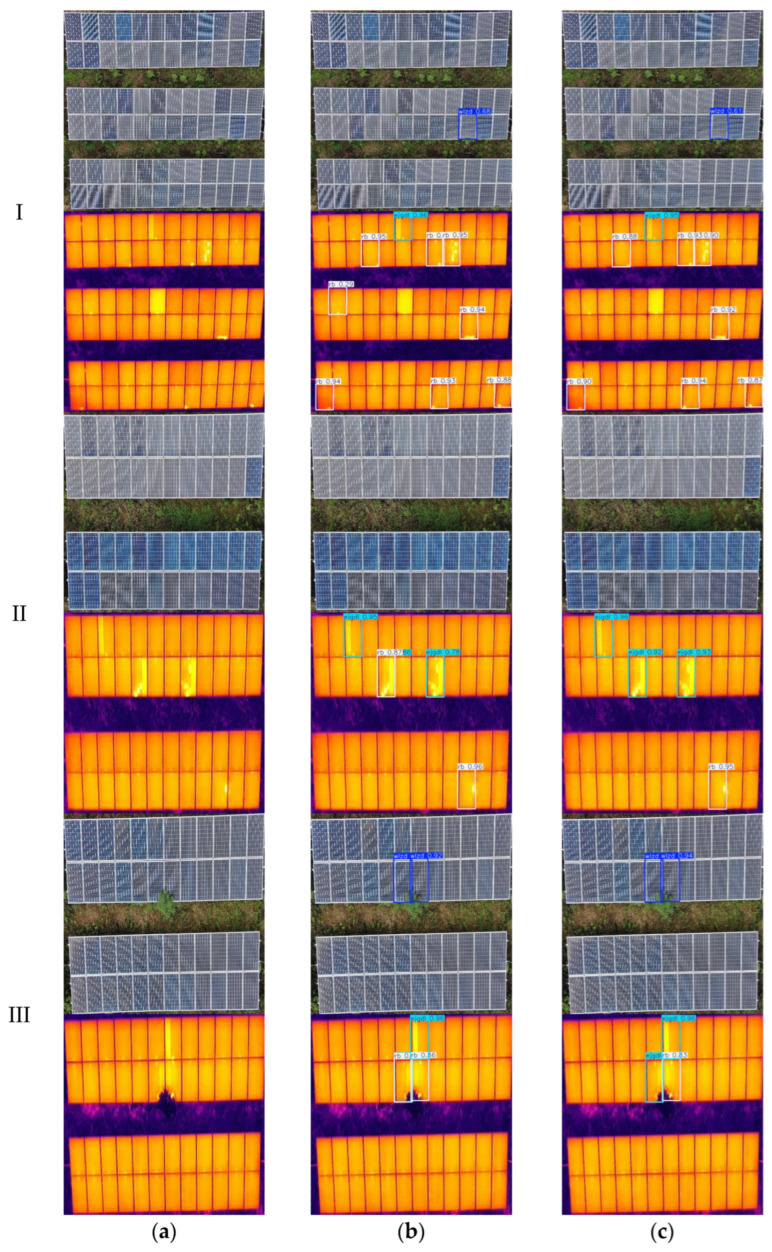
Photovoltaic module fault testing diagram. (**a**) Original image, (**b**) Mid-fusion-YOLOv11n, (**c**) RFE-YOLO. I–III indicate three different example cases.

To evaluate the overall performance of the improved RFE-YOLO model, three representative sets of visible and infrared images were selected from the test dataset for comparative analysis. The detection results before and after model enhancement are shown in Figure 14.

In testing three sets of fault images from different types of photovoltaic modules, this study compares the mid-term fusion YOLOv11n model with the improved RFE-YOLO model. The experiments included visible light images containing only physical obstruction categories, while infrared images involved only hot spots and diode short circuits. Overall test results indicate that the RFE-YOLO model achieves more accurate PV module fault identification. In Group I images, the mid-term fusion YOLOv11n model misclassified normal areas as hot spot faults in infrared images, whereas the RFE-YOLO model accurately distinguished them and produced higher confidence detection outputs for other faults. In Group II images, both models correctly identified the absence of physical shading in visible light images. However, in infrared images, the mid-fusion YOLOv11n model misclassified diode short-circuit faults as hot spot faults, whereas the RFE-YOLO model not only correctly identified the fault type but also demonstrated significantly higher confidence levels than the comparison model. In Group III images, both models detected physical obstruction in visible light images, but the RFE-YOLO model demonstrated markedly higher detection confidence. In infrared images, the mid-term fusion YOLOv11n model again misclassified diode short-circuit faults as hot spots, whereas the RFE-YOLO model achieved accurate identification.

In summary, the RFE-YOLO model outperforms the mid-term fusion YOLOv11n model in both fault detection accuracy and confidence levels, effectively reducing false detections. This result demonstrates that the improved RFE-YOLO model exhibits greater robustness and reliability in photovoltaic module fault detection tasks.

## 5. Discussion

Rapid and accurate detection of photovoltaic module failures is crucial for power plant health management and operational decision-making. To this end, this study constructs the multimodal detection model RFE-YOLO for visible-infrared images based on the lightweight and deployable YOLOv11n framework. By comparing different fusion strategies, feature-level mid-term fusion is selected as the multimodal fusion architecture. The model’s core introduces a multi-modal re-parameterized feature extraction RC module based on the CBAM attention mechanism. To address the imbalance in dual-modal quality caused by environmental variations, heterogeneous RC_V and RC_I submodules are designed to differentially enhance visible light structural features and infrared thermal distribution features. A lightweight FA fusion module is further introduced, which adaptively integrates complementary information through learnable modality balancing and cascaded attention mechanisms. The GSConv-constructed EVG enhancement module strengthens the extraction of shallow textures and deep semantics while ensuring computational efficiency, significantly improving detection accuracy and robustness for subtle defects such as hot spots and diode short circuits.

Although the model demonstrates strong performance under controlled dataset conditions, its feature representation and prediction stability require improvement when addressing imaging quality degradation caused by sudden environmental changes, such as overexposure, low contrast, and noise interference. Future research should integrate edge computing devices with IoT cloud platforms to systematically validate the model’s real-time performance in multimodal data scenarios, ensuring its robustness and reliability in complex real-world environments. In terms of model accuracy and related metrics, this study achieves significant improvements over existing methods. However, experimental results also reveal several pressing issues. As a crucial foundation for model experimentation, although this paper strives to simulate real-world detection tasks for photovoltaic modules, limitations remain, including restricted target category coverage and inconsistent data quality. Furthermore, uncertainties introduced during manual annotation may lead to labeling errors or classification biases, potentially affecting model training outcomes. Regarding model architecture optimization, while RFE-YOLO has made phased progress in parameter control and computational complexity, further exploration of lighter network structures and algorithms is needed to enhance computational efficiency while maintaining high accuracy. Through continuous refinement of model design, more efficient computational performance in practical applications is anticipated, accelerating the deployment of related technologies. In the context of photovoltaic power plant operations and maintenance, this study offers a practical and lightweight solution for module fault detection. It assists maintenance personnel in rapidly identifying component failures, reduces reliance on manual inspections, and enhances fault response efficiency. It is hoped that this work will contribute to advancing intelligent monitoring technologies for power plant operations and maintenance.

## 6. Conclusions

This paper constructs an RGBIRPV dataset based on real-world centralized PV power plant environments, featuring matched infrared and visible light images. This enriches data resources in the field and provides crucial support for in-depth research on PV module fault detection tasks. Based on this dataset, we developed an RFE-YOLO detection model tailored for multimodal PV module images. The model’s core comprises three synergistic modules: an RC feature extraction module designed with CBAM attention and reparameterization techniques, where heterogeneous RC_V and RC_I submodules enhance visible structural textures and infrared thermal distribution features, respectively, effectively mitigating modal quality imbalance caused by imaging condition variations; We propose a lightweight adaptive fusion FA module that dynamically adjusts the contribution of dual-modal features through learnable weight allocation and attention cascading mechanisms, achieving efficient integration of complementary information. We introduce an EVG multi-scale enhancement module based on GSConv, which maintains low computational overhead while strengthening the fusion of shallow details and deep semantics, significantly improving robustness in identifying minute defects such as hot spots and diode short circuits.

Experimental results demonstrate that the proposed model achieves a mAP of 83.7% on the real-world test dataset. Compared to the baseline method, it improves mAP by 1.8% and precision by 2.9%, while reducing the number of parameters and computational complexity by 3.2% and 5.4%, respectively. These findings validate the effectiveness of this approach for multimodal photovoltaic module fault detection, providing a feasible technical pathway for real-time intelligent monitoring of power plants.

Future research will focus on three key areas: (1) Integrating historical power generation performance data with multimodal UAV imagery to construct a joint dataset covering a broader range of failure modes, while exploring deep learning-based predictive diagnostic methods for precise severity assessment; (2) Further reducing computational overhead through techniques such as model pruning, quantization, or knowledge distillation, and investigating ultra-lightweight architectures suitable for resource-constrained edge devices; (3) Promote the practical integration and field deployment of RFE-YOLO within photovoltaic power plant operation and maintenance systems to enable dynamic early warnings for module status, precise fault localization, and effective control.

## Figures and Tables

**Table 1:** Experimental Environment and Parameter Settings.

Configuration Name	Configuration Parameters
Operating System	Windows 10
CPU	i9-10900K
GPU	NVIDIA RTX 4090
Memory	128G
Software	Pycharm2024
Python	3.8
PyTorch	2.2.0
CUDA	11.8

**Table 2:** Key Parameter Settings.

Parameter	Value
Learning Rate	0.01
Image Size	640 × 640
Momentum	0.937
Optimizer	SGD
Batch Size	16
Epoch	200
Weight Decay	0.0005

**Table 3:** Detection Performance Comparison of Different Fusion Strategies in the YOLOv11 Network.

Model	*p*	R	mAP@50	mAP@50:95	F1	Para(M)	GFLOPs
Pixel-level fusion	0.791	0.727	0.811	0.649	0.758	2.6	6.5
Feature-level early fusion	0.808	0.726	0.804	0.649	0.765	4.3	10.7
Feature-level mid-fusion	0.796	0.763	0.819	0.667	0.779	3.9	9.5
Decision-level integration	0.782	0.766	0.813	0.613	0.774	5.1	12.6

**Table 4:** Results of the 5-fold cross-validation experiment.

Fold	*p*/%	R/%	mAP@50/%	F1/%
Fold 1	81.2	76.4	82.0	78.7
Fold 2	81.7	73.6	80.8	77.4
Fold 3	83.5	76.3	83.4	79.7
Fold 4	80.5	78.1	82.5	79.3
Fold 5	79.5	79.2	83.0	79.4
Average Standard	81.3	76.7	82.3	78.9
deviation	1.5	2.1	1.0	0.8

**Table 5:** Comparison of the experimental results.

Fold	*p*/%	R/%	mAP@50/%	F1/%
Fold 3	83.5	76.3	83.4	79.7
deviation	80.1	76.7	83.2	78.3

**Table 6:** Compression Channel Parameter Selection.

RC_V	RC_I	*p*/%	R/%	mAP@50/%	F1/%
8	8	78.5	77.2	81.8	77.8
8	16	80.7	75.4	82.8	77.9
16	8	83.8	74.1	83.0	78.6
16	16	81.2	76.3	82.3	78.6

**Table 7:** Parameter Selection for Heterogeneous Convolution Kernels.

RC_V	RC_I	*p*/%	R/%	mAP@50/%	F1/%
3	3	78.0	77.9	81.5	77.9
3	5	83.8	74.1	83.0	78.6
5	3	81.5	75.5	82.3	78.3
5	5	79.2	77.9	82.3	78.5

**Table 8:** Ablation experimental results.

Model	RC	FA	EVG	*p*/%	R/%	mAP@50/%	mAP@50:95/%	F1/%	Params/10^6^	GFLOPs	Model Size/MB
A				79.6	76.3	81.9	66.7	77.9	3.95	9.5	8.3
B	√			83.8	74.1	83.0	67.1	78.7	3.72	9.5	8.0
C		√		81.5	76.0	82.5	66.9	78.7	3.83	9.2	8.1
D			√	82.6	74.3	82.7	67.1	78.2	3.65	9.3	7.8
E	√	√		82.4	75.6	83.3	67.2	78.9	3.61	9.2	7.8
F	√		√	81.3	77.4	83.4	67.1	79.3	3.74	10.1	8.1
G		√	√	80.3	77.7	83.3	67.5	79.0	3.59	8.7	7.7
H	√	√	√	82.5	76.5	83.7	67.6	79.4	3.69	9.2	8.0

Note: A is the feature-level mid-fusion YOLOv11n model; B is the feature-level mid-fusion YOLOv11n + RC model; C is the feature-level mid-fusion YOLOv11n + FA model; D is the feature-level mid-fusion YOLOv11n + EVG model; E is the feature-level mid-fusion YOLOv11n + RC + FA model; F is the feature-level mid-fusion YOLOv11n + RC + EVG model; G is the feature-level mid-fusion YOLOv11n + FA + EVG model; H is the RFE-YOLO model.

**Table 9:** Comparative experiment.

Model	*p*/%	R/%	mAP@50/%	mAP@50:95/%	F1/%	Params/10^6^	GFLOPs	Model Size/MB
YOLOv5n (IR)	75.0	77.6	80.2	58.8	76.3	2.50	7.1	5.3
YOLOv6n (IR)	77.5	74.8	79.9	57.4	76.1	4.23	11.8	8.7
YOLOv8n (IR)	77.7	75.8	80.1	59.5	76.7	3.00	8.1	6.3
YOLOv9t (IR)	77.7	75.9	80.4	58.3	76.8	1.97	7.6	4.5
YOLOv10n (IR)	79.3	73.0	80.2	59.4	76.0	2.77	8.2	5.5
YOLOv11n (IR)	77.0	77.1	80.6	60.8	77.0	2.58	6.3	5.5
YOLOv12n (IR)	80.0	74.1	79.9	58.9	76.9	2.50	5.8	5.3
RT-DETR-l (IR)	78.4	75.3	79.6	65.0	76.8	31.98	103.4	66.2
Mid-fusion-YOLOv11n (RGB + IR)	79.6	76.3	81.9	66.7	77.9	3.9	9.5	8.3
DEYOLO (RGB + IR)	81.0	74.3	82.1	65.3	77.5	5.99	9.7	12.4
ICAFusion (RGB + IR)	79.9	75.3	79.2	64.2	77.5	7.05	10.2	14.6
RFE-YOLO (RGB + IR)	82.5	76.5	83.7	67.6	79.4	3.69	9.2	8.0

## Data Availability

The data presented in this study are available on request from the corresponding author. The data are not publicly available due to ongoing study.
